# Global transcriptome analysis of *Mesorhizobium alhagi* CCNWXJ12-2 under salt stress

**DOI:** 10.1186/s12866-014-0319-y

**Published:** 2014-12-24

**Authors:** Xiaodong Liu, Yantao Luo, Osama Abdalla Mohamed, Dongying Liu, Gehong Wei

**Affiliations:** State Key Laboratory of Soil Erosion and Dryland Farming on the Loess Plateau,, College of Life Sciences, Northwest A&F University, Yangling, Shaanxi 712100 China; Institute for Post Graduate Environmental Studies, Environmental Science Department, Suez Canal University, El-Arish Branch, 45511 Ismailia, Egypt

**Keywords:** Salt stress, RNA-Seq, Secretion system, Chaperones, *Mesorhizobium alhagi*

## Abstract

**Background:**

*Mesorhizobium alhagi* CCNWXJ12-2 is a α-proteobacterium which could be able to fix nitrogen in the nodules formed with *Alhagi sparsifolia* in northwest of China. Desiccation and high salinity are the two major environmental problems faced by *M. alhagi* CCNWXJ12-2. In order to identify genes involved in salt-stress adaption, a global transcriptional analysis of *M. alhagi* CCNWXJ12-2 growing under salt-free and high salt conditions was carried out. The next generation sequencing technology, RNA-Seq, was used to obtain the transcription profiles.

**Results:**

We have compared the transcriptome of *M. alhagi* growing in TY medium under high salt conditions (0.4 M NaCl) with salt free conditions as a control. A total of 1,849 differentially expressed genes (fold change ≧ 2) were identified and 933 genes were downregulated while 916 genes were upregulated under high salt condition. Except for the upregulation of some genes proven to be involved in salt resistance, we found that the expression levels of protein secretion systems were changed under high salt condition and the expression levels of some heat shock proteins were reduced by salt stress. Notably, a gene encoding YadA domain-containing protein (*yadA*), a gene encoding trimethylamine methyltransferase (*mttB*) and a gene encoding formate--tetrahydrofolate ligase (*fhs*) were highly upregulated. Growth analysis of the three gene knockout mutants under salt stress demonstrated that yadA was involved in salt resistance while the other two were not.

**Conclusions:**

To our knowledge, this is the first report about transcriptome analysis of a rhizobia using RNA-Seq to elucidate the salt resistance mechanism. Our results showed the complex mechanism of bacterial adaption to salt stress and it was a systematic work for bacteria to cope with the high salinity environmental problems. Therefore, these results could be helpful for further investigation of the bacterial salt resistance mechanism.

**Electronic supplementary material:**

The online version of this article (doi:10.1186/s12866-014-0319-y) contains supplementary material, which is available to authorized users.

## Background

*Mesorhizobium alhagi* CCNWXJ12-2, was classified as a high salt-tolerant rhizobium, which could be able to form nitrogen-fixing symbiosis with desert plant *Alhagi sparsifolia* [1]. *Alhagi sparsifolia* has a large distribution in the southern fringe of Taklamakan Desert and it plays an important role in windbreaker and sand fixation and social-economy at that area [2]. Undoubtedly, salinity and desiccation, are severe problems facing the agricultural industry due to cases the degradation of soil quality [3]. Almost 40% of the world’s land surface are troubled by salinity [4]. These condition has detrimental effects on the Rhizobium-Legume symbiosis in different ways such as the growth and survival of rhizobia in soil colonization around the root and interaction between rhizobia and legumes or functions of the nodules [5]. In order to form efficient nitrogen-fixing symbiosis, the rhizobia should have high salt resistance to be able to survive in salty soils. Therefore, in recent years the number of studies on salt-tolerant rhizobia and the mechanism of salt resistance are increasing. In particular, most of the articles focus on the isolation of salt-tolerant rhizobia or some specific genes involved in salt resistance [6-10]. Nevertheless, the salt resistance mechanism in rhizobia is still complicated and need a lot of efforts to elucidate it.

During the last decade, DNA array technology was widely used to identify the differentially expressed genes of bacteria cultured under different condition [11-13]. In recent years, the next generation sequencing technology RNA-Seq has been wildly used in transcriptome analysis in bacteria [14]. Therefore, this kind of sequencing technology has been used to find the functional elements of the genome, the differentially expressed genes under different conditions and the new noncoding RNAs [15-17]. The objectives of the present work were to investigate the transcriptional changes in rhizobia subjected to long-term exposure to high concentration of NaCl and to make a transcriptome analysis of *Mesorhizobium alhagi* CCNWXJ12-2 during late exponential growth at two different concentrations of NaCl (0 and 0.4 M) by using the next generation sequencing technology RNA-Seq. The results showed a series of genes differentially expressed during long-term salt stress, among which were genes encoding an outer membrane adhesion protein (YadA), a co-methyltransferase (MttB) and a formate-tetrahydrofolate ligase (Fhs). Gene knockout and comparative growth analysis indicated that *yadA* was involved in salt resistance directly but the other two genes were not.

## Results and discussions

### Experimental design and global overview of the RNA-Seq data

The genome of *Mesorhizobium alhagi* strain CCNWXJ12-2 has been reported in 2012 [1] and the growth curves of *Mesorhizobium alhagi* under different salt concentrations (0, 0.1, 0.2, 0.3, 0.4, and 0.5 M of NaCl) were measured (Additional file 1). The growth of *Mesorhizobium alhagi* under 0.5 M of NaCl was dramatically delayed while under 0.4 M was acceptable. So we chose 0.4 M of NaCl as the high-salt condition. According to the salt tolerance research of the other mesorhizobium type strains (most sensitive to 0.26 M NaCl), *Mesorhizobium alhagi* possessed a relatively high salt resistance [18]. To investigate the salt resistance mechanism of *M. alhagi* under long-term salt stress, we have decided that to conduct a global transcriptome analysis of the strain growing under salt-free and high-salt conditions (0.4 M NaCl) at late exponential phase. Total RNA was extracted from three independent biological replicates and then mixed together for RNA sequencing. A total of 5,942,210 and 5,987,420 reads were obtained from control and salt-treatment condition, respectively. After filtration, the low quality reads 5,640,246 clean reads of control and 5,627,881 of salt-treatment were retained. The average length of the reads was 100 bp. For each of the two samples >95% of all reads was mapped to the reference genome.

**Figure 1:**
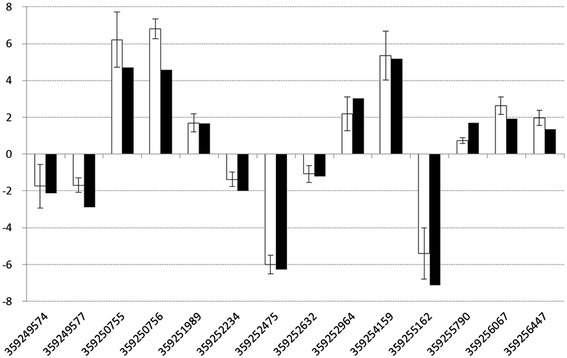
**Validation of RNA-Seq data using RT-qPCR.** Fourteen representative genes were chosen to validate the RNA-Seq data by RT-qPCR. The white bars represent mean values of log2-transformed fold change obtained from three biological replicates of RT-qPCR with error bars stand for standard deviations. And the black bars represent RNA-Seq data.

The RNA-Seq data were validated by analyzing 14 representative genes expression levels using RT-qPCR. The log2-transformed mean values for each gene of three biological replicates were in good agreement with the log2-transformed fold change of RNA-Seq (Figure 1).

### Gene expression patterns under high-salt and salt-free conditions

RNA-Seq data showed that most of the predicted genes were expressed under both cultivating conditions. Among 7,407 predicted protein coding genes in the reference genome, there were 5,495 (74.19% of the total genes, RPKM > =20) and 5,459 (73.7% of the total genes, RPKM > =20) genes were detected in control and salt-treatment condition, respectively.

**Figure 2:**
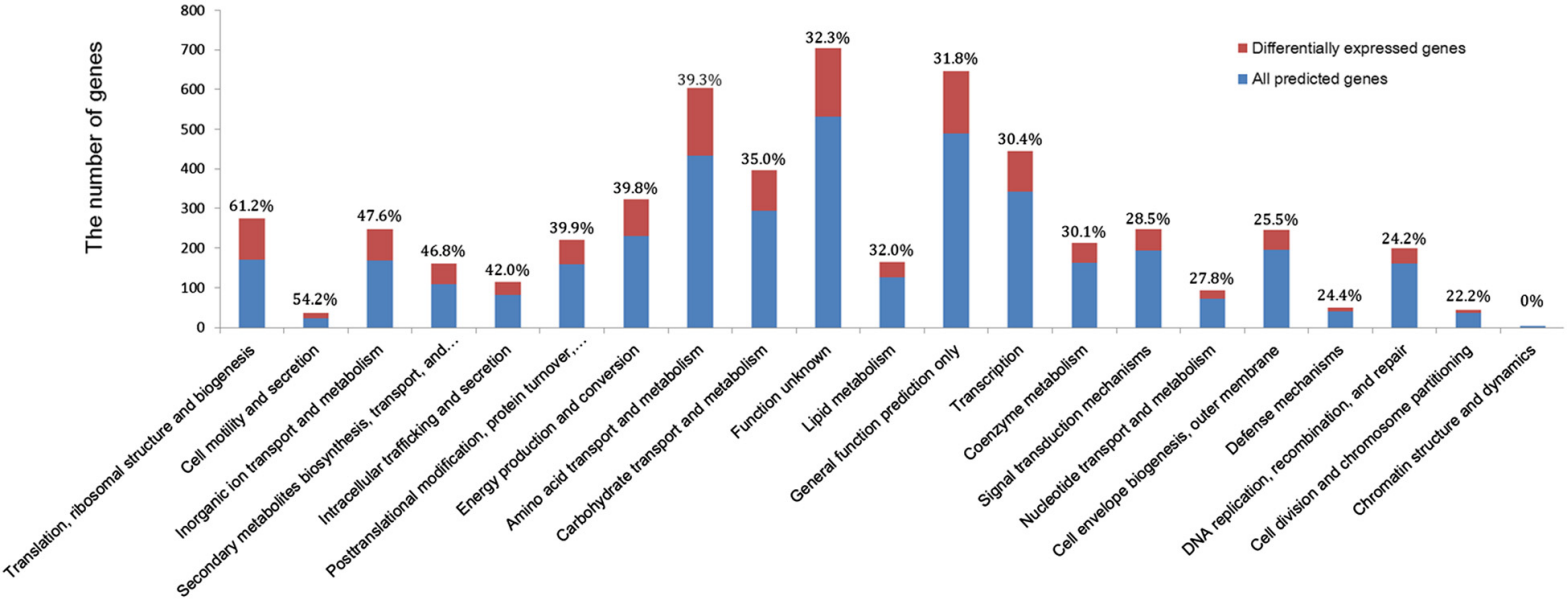
**The COG categories of all predicted genes and the DEGs.** Summary of COG annotations for all the predicted genes and the number of differentially expressed genes in each COG category. The percentage of the the DEGs account for the predicted genes were shown up the bars.

**Figure 3:**
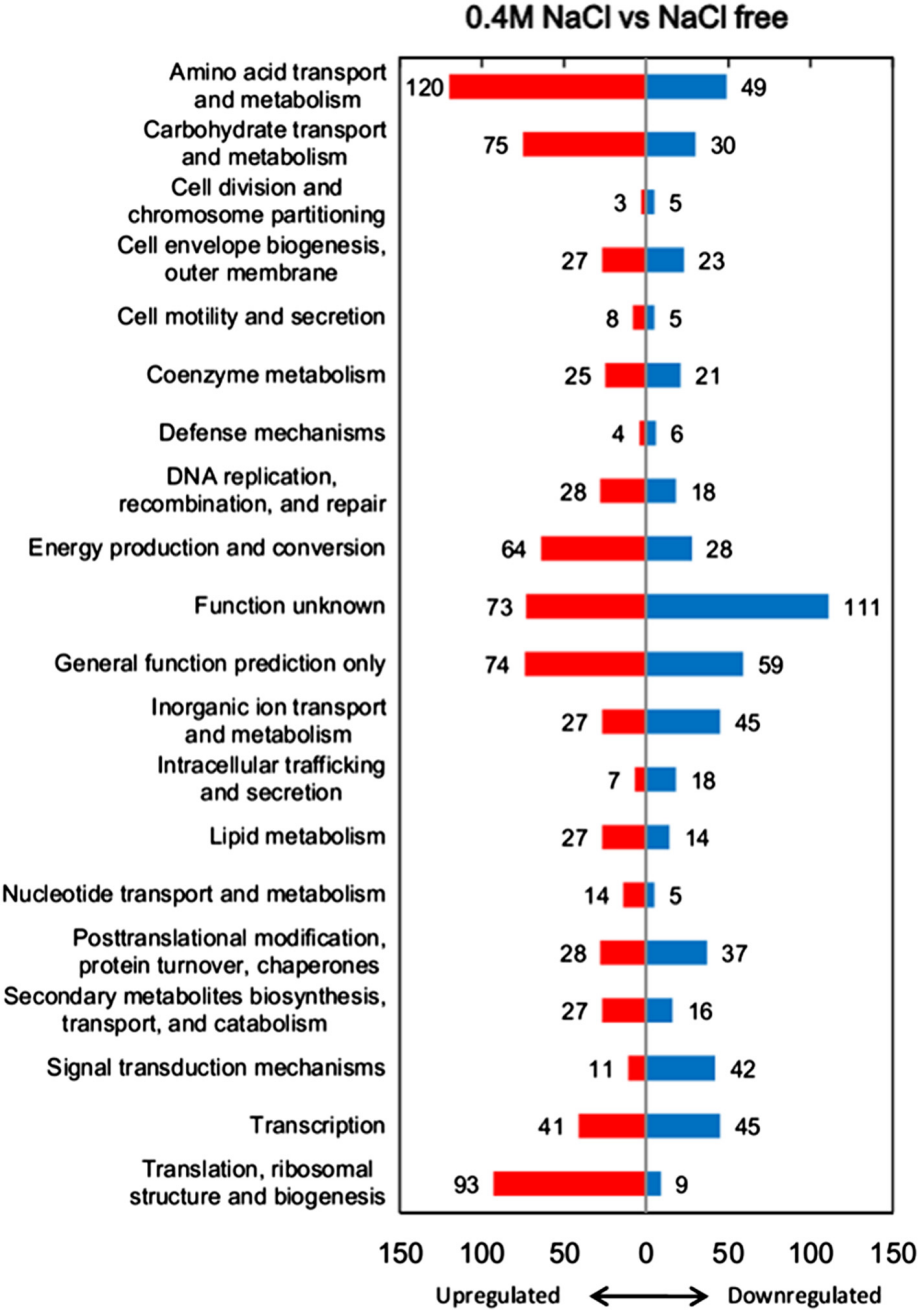
**The numbers of the DEGs grouped by functional categories.** The red and blue bars represent up- and down-regulated genes, respectively, and the numeric lables represent the number of genes in the function.

Cluster of orthologous groups (COG) is widely used to classify orthologous proteins [19]. Based on the conserved region, every protein is assumed to be evolved from an ancestor protein in COG database. Thus, this database is helpful to annotate the genes from poorly characterized genomes [20]. Aligned all of the predicted transcribed (RPKM > =20) genes against the COG database 4,022 genes obtained their COG codes. The Figure 2 presents the COG categories of all the proteins encoded by transcribed genes and the DEGs and the number of each category are shown in Additional file 2. The DEGs account for high ratios in most COG categories, which means the salt resistance mechanism in *Mesorhizobium alhagi* should be complex. Furthermore, among the total 1,849 differentially expressed genes (DEGs), 933 and 916 were significantly down-regulated and up-regulated response to salt stress, respectively. Consequently, this large number of DEGs suggested that not only the salt-specific stress responses but also the nonspecific responses were triggered. DEGs were then grouped by functional categories (Figure 3). Indeed, there are many genes involved in amino acid transport and metabolism, carbohydrate transport and metabolism, energy production and conversion and translation were induced by salt stress. The 184 genes of function unknown, 133 genes of general function predicted and 487 genes without COG codes indicated that *Mesorhizobium alhagi* XJ12-2 might have some unknown means to deal with the high salt condition.

The Kyoto Encyclopedia of Genes and Genomes (KEGG) Pathway database collects all known networks of molecular interactions in different species. The pathway analysis of the DEGs could be helping us to understand the interactions of these genes. With compared 7,407 predicted genes against the KEGG database using BBH method [21], a total of 2,568 putative proteins obtained their KO (KEGG Orthology) codes. The pathway enrichment analysis was conducted to make better understanding of how the *Mesorhizobium alhagi* adpat to salt stress. The threshold of FDR (false discovery rate) was setted to 0.001. The resutls showed that pathway enrichment of the downregulated genes was located in the KO term of Membrane Transport while the pathway enrichment of the upregulated genes was located in the KO terms of Transcription and Translation (Additional files 3 and 4). The results indicated that the protein synthesis enhanced during long-term salt stress.

### DEGs involved in adaption to salt stress

Challenged with reduction in external water activity, the bacteria could be accumulating osmoprotectants to alleviate the inhibitory effects caused by high osmolarity [22]. The osmoprotectants include many macromolecules such as glycine betaine (GB), proline, and some other structurally related zwitterionic molecules [23]. Our RNA-Seq data showed that the expression level of genes involved in GB/proline uptaking *(proVWX*) was induced significantly by salt stress at 0.4 M NaCl and the *proX* showed a 35.9-fold up-regulated (Table 1). In *Rhodobacter sphaeroides f. sp. Denitrificans* IL106, trehalose is also another important osmoprotectant play an important role in salt resistance [24]. Our data showed that the gene expression level of trehalose synthase (*treT*) was repressed by high salt while the gene expression level of other two trehalose 6-phosphate synthases (otsA) were upregulated slightly (Table 1). The expression level of the Na^+^/H^+^ ions antiporter gene nhaA was also upregulated (Table 1). Na^+^/H^+^ ions antiporters play an important role in keeping ion homeostasis in many organisms [25]. In case of *E. coli*, *nhaA* made a great contribution to maintaining intracellular pH and Na^+^ homeostasis [26]. Whereas, in *Vibrio cholera*, *nhaA* was required for the bacteria to survive in a saline environment [27]. Some reports investigated the expression of exogenous *nhaA* in yeast and rice enhanced their salt tolerance [28,29]. Our results showed that most of the genes proven to be related to salt resistance in other organisms were also upregulated in *M. alhagi* growing under high salt condition (Table 1). Based on this point, we considered that the uptaking of osmoprotectant and the high expression of ion transporter genes were important means to cope with high salinity in *M. alhagi*.

**Table 1:** **DEGs involved in salt resistance**

**GI number** ^**a**^	**Log2(Fold_change) normalized**	**P-value**	**Gene**	**Gene discreption**
359254159	5.168	0	*proX*	glycine betaine transporter periplasmic subunit
359254161	3.539	1.22E-286	*proV*	glycine betaine/L-proline ABC transporter ATPase subunit
359254160	1.672	5.93E-16	*proW*	glycine betaine/L-proline transport system permease protein
359254492	1.665	7.04E-170	*proX*	choline ABC transporter periplasmic protein
359256447	1.361	5.51E-39	*nhaA*	Na+/H+ antiporter NhaA
359254494	1.455	8.30E-23	*proV*	ABC transporter ATP-binding protein
359254493	1.341	1.24E-11	*proW*	glycine/betaine/proline ABC transporter membrane protein
359251246	−1.485	7.30E-64	*treT*	trehalose synthase
359256049	0.379	0.000108	*otsA*	trehalose 6-phosphate synthase
359248822	0.371	0.133699	*otsA*	trehalose 6-phosphate synthase

^a^GI number, GenInfo Identifier.

### DEGs of other processes

Our results showed that many genes involved in cell growth, protein synthesis and energy production were upregulated under high salt condition (Additional file 5). The upregulation of these genes might suggest that the cell density of *M. alhagi* was higher under high salt conditions (0.4 M NaCl) at late exponential phase than that under no salt conditions (0 M NaCl). In recent years, there are some reports showed the expression of genes involved in cell growth, protein synthesis and energy production were upregulated in some microorganisms growing under high salt conditions[30,31]. In *Staphylococcus sp*. OJ82, isolated from high salt resistance fermented seafood, the expression level of genes involved in energy production, translation and cell membranes synthsis was induced by salt stress [30]. The expression level of *fabG*, a gene involved in biosynthesis of unsaturated fatty acids was highly upregulated by salt stress in *Synechocystis sp*. PCC 6803 [31]. Our RNA-Seq data, *fabG*, *F*, *I*, *H* and *D*, genes involved in unsaturated fatty acids biosynthesis, were all upregulated significantly except for *fabB*. According to our results we suggested that the bacteria needed to produce more energy and functional proteins to cope with the unsatisfied environmental conditions and the upregulation of these genes may also explain that the biomass of *M. alhagi* growing under high salt conditions was more than that growing in TY without NaCl (Additional file 5).

Besides that the genes were involved in cell growth, we still found a significant change in protein secretion systems (Additional file 6). There are two type III secretion systems (T3SSs) operons in *M. alhagi* CCNWXJ12-2, designated T3SS1 (359250064–359250082) and T3SS2 (359251150–359251163). So, our results showed that the expression level of T3SS1 increased under high salt conditions while T3SS2 was decreased (Additional file 6). In some bacteria, the expression levels of the T3SS genes were affected by salt. In *Pseudomonas aerug*inosa, T3SS was upregulated at steady-state osmotic stress while it was downregulated during osmotic up-shock [32]. In *Yersinia enterocolitica Biovar* 1B, the Ysa T3SS was expressed when the concentration of NaCl in medium was greater than 180 mM and reached to the maximal level when the concentration of NaCl reached 290 mM [33]. In some rhizobium, the T3SS was involved in the interactions between the bacteria and the legumes plant to form the nodules [34-36]. We also found that the type VI secretion system (T6SS) and type IV secretion system (T4SS) were downregulated in the presence of 0.4 M NaCl (Additional file 6). Noteworthy, T6SS was not only involved in pathogenicity in some pathogens but also involved in keeping intracellular balance of H^+^ ions in *Yersinia pseudotuberculosis* [37-39]. Whereas, recent research papers showed that the T6SS was also involved in interbacterial interactions and competition between different bacteria genera [40,41]. In *Rhizobium leguminosarum bv. trifolii*, the T6SS was involved in the interaction between bacteria and legumes after the rhizobia inoculated to the plants [42,43]. In many bacteria T4SSs were involved in conjugal transfer of DNA [44]. However, in *Mesorhizobium loti strain R7A*, T4SS functioned as T3SSs of other rhizobium and had a role in symbiosis [45]. But in contrast to *M. loti*, T4SS in *Sinorhizobium meliloti strain* 1021 had no function in host invasion and symbiotic formation [46]. Besides the protein secretion systems, we still found two lytic transglycosylases (LTs,359253896 and 359252475) downregulated under salt stress (Additional file 6). LTs are a family of enzymes that could cleave glycosydic bonds within peptidoglycan sacculus to make space for some processes occurred within the cell envelope or assembly and anchoring of secretion systems [47,48]. The LTs also had functions in cell division and the downregulation of these enzymes might be explain the delay of the cell growing under high salt conditions and consist with the downregulation of the protein secretion systems [48]. Up to a point, the changes of the expression levels of the T3SSs, T6SS, T4SS and LTs genes under high salt condition we hypothesized that the environmental factor of high salt might be have influences on the communication between *M. alhagi* CCNWXJ12-2 and plants or other bacteria.

The RNA-Seq data showed that the molecular chaperones *GroES*, *GroEL*, *DnaK*, *DnaJ* and *ClpB* were all downregulated (Table 2). The risk of proteins unfolding was increased when organisms cultured under environmental stresses and these molecular chaperones could be help the proteins fold properly and reliably [49]. In this regard, it was very interesting of the downregulation of these genes under high salt conditons. In previous research, the heat shock proteins from *E. coli* and *B. subtilis*, *DnaK* or *GroEL* were not induced by supplement of salt in the medium [50-52]. But researchers also found that *DnaK* was involved in K^+^ ions transport at high osmolarity in *E. coli* [53]. In *Rhizobium tropici* DIAT899 the *DnaJ* insertion mutant was showed sensitivity to salt stress [54]. ClpB is a common molecular chaperone which could interact with DnaK and catalyze the proteins disaggregation and reactivation [55]. Transcriptome analysis of *Staphylococcus* sp. OJ82 growing under high salt condition showed that the expression of some molecular chaperones were downregulated, too [30]. We are confused why the expression of the molecular chaperones was downregulated when *M. alhagi* grown under high salt conditions. Therefore, the functions of these molecular chaperones in *M. alhagi* should be studied to explain this phenomenon. Because the transcriptome analysis just reflected the changes of mRNA level, so we suggested that the proteins levels of these genes should be investigated.

**Table 2:** **DEGs of molecular chaperones**

**GI number**	**log2(Fold_change) normalized**	**P-value**	**Gene**	**Gene function**
359252555	−1.006	7.17E-34	*groEL*	chaperonin GroEL
359252235	−2.494	2.68E-103		co-chaperonin GroES
359255864	−1.155	7.59E-134	*dnaK*	molecular chaperone DnaK
359250220	−1.989	3.11E-118		heat shock protein DnaJ domain-containing protein
359254957	−2.742	0		ATP-dependent chaperone ClpB

### Characterization of gene knockout and complementation mutants

**Figure 4:**
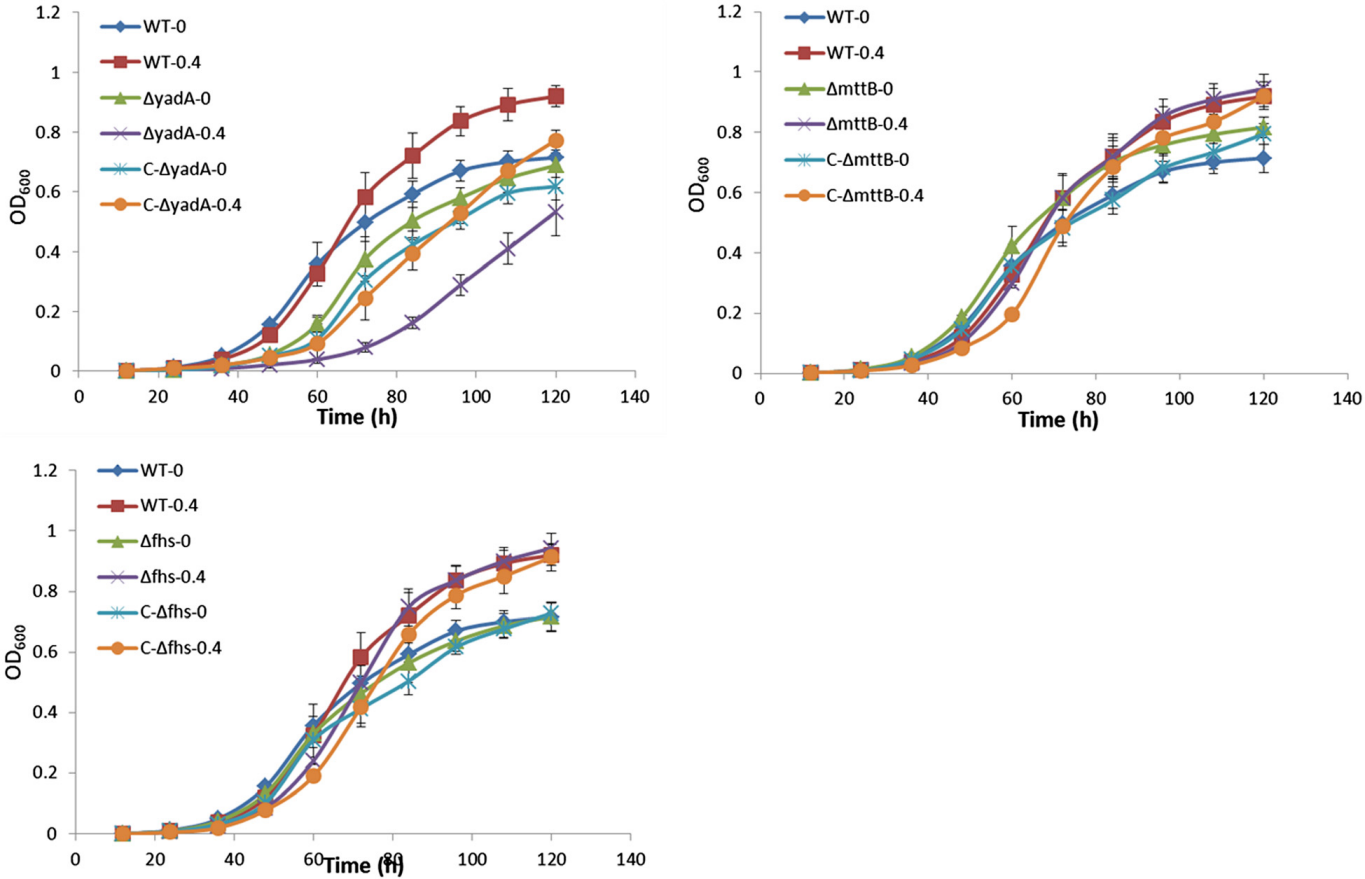
**Growth curves of the wild type and mutants.** Optical density at 600 nm(OD_600_) was used to monitor the bacteria growth. Three independent biological experiments were conducted to measure the growth of the wild type *M. alhagi* and the mutants. The error bars stand for standard deviations. A *yadA* mutant and the complementation mutant; B *mttB* mutant and the complementation mutant; C *fhs* mutant and the complementation mutant.

To identify the involvement in salt response, three genes highly induced by salt stress (*yadA*, *mttB* and *fhs*) were selected for gene mutant construction since so far no reports show that they are involved in salt resistance. The expression data of the three genes were listed in Table 3. In order to study of *yadA* (Yersinia adhesin A), researchers focus on the importance of the gene function on the pathogenicity of *Yersinia* and this gene was found to be a major virulence factor of *Yersinia enterocolitica* [56-58]. YadA is a trimeric autotransporter produced by *Yersinia enterocolitica* and it has manifold functions such as helping bacteria adhesion to host cells, promoting autoaggregation and protection of the bacteria from complement-mediated killing [59]. Gene *mttB* was encoding a co-methyltransferase which involved in the methane metabolism in *Methanosarcina* and its function in *Mesorhizobium* is still unknown [60,61]. Gene *fhs* was encoding a formate-tetrahydrofolate ligase involved in the biosynthesis of purine, Met-tRNA, methionine, serine and some other amino acids in *Streptococcus sp* [62]. The upregulation of the *fhs* consisted with the upregulation of genes involved in protein synthesis. The growth curves of the mutants in TY broth medium under salt stress showed that the growth rate of mutant Δ*yadA* was delayed under high salt condition while the growth of the other two mutants were almost the same with the wild type (Figure 4). The growth curve of Δ*yadA* complementation mutant showed a restored salt tolerance, although it cannot reach the wild type level (Figure 4). This may be caused by the activity of the plasmid promoter was not strong in *Mesorhizobium alhagi*. In Figure 4, the delay of the growth under high salt condition was caused by the different culture conditions. Thus, we considered that gene *yadA* was involved in salt resistance while the other two genes were not.

**Table 3:** **Gene expression of genes** ***yadA*** **,** ***mttB*** **and** ***fhs***

**Gene**	**log2(Fold_change) normalized**	**P-value**	**Gene function**
***yadA***	4.567258	0	YadA domain-containing protein
***mttB***	3.771452	2.38E-213	Trimethylamine methyltransferase
***fhs***	3.758628	0	Formate--tetrahydrofolate ligase

## Conclusion

In our study, we have used RNA-Seq to obtain the transcriptome profiles of *M. alhagi* CCNWXJ12-2 growing under high salt and salt-free conditions and compared them to try to elucidate the mechanism of salt resistance. Our results showed that the expression of many validated genes involved in salt resistance in other bacteria was also induced by high salt in *M. alhagi*, such as *proV*, *proW*, *proX* and *nhaA*. Moreover, there are many genes involved in cell growth, energy production and translation were also upregulated by salt stress. Based on our results, we consider that the osmoprotectants uptaking and the ion transporters are the two important ways to cope with the salt stress in *M. alhagi*. The enhanced energy production and protein synthesis as well as the decreased protein secretion systems and other unimportant processes could also improve the ability of the bacteria to survive under high salt condition. To our knowledge, this is the first report about transcriptome analysis of a rhizobia using RNA-Seq to elucidate the salt resistance mechanism and we believe that our results could be consider as a reference work for the further salt resistance researches.

## Methods

### Bacterial strains and growth conditions

*Mesorhizobium alhagi* CCNWXJ12-2 was used for all the experiments. The genome of this strain was sequenced and published in 2012 [1]. Single colonies were selected and checked for purity by repeated streaking and microscopic examination. All isolates were incubated at 28°C and maintained on TY agar plate (5 g tryptone, 3 g yeast extract, and 0.7 g CaCl_2_ · 2H_2_O per liter). Strain CCNWXJ12-2 was pre-cultured in 10 ml TY broth medium. The cultures were incubated at 28°C and agitated at 180 rpm for 3 days. The growth values of the strains were determined by absorbance at 600 nm (OD_600_). One milliliter of the CCNWXJ12-2 suspension was pre-cultured into 100 ml TY broth medium supplemented with 0.4 M NaCl also at the same time a control experiment was conducted. At the end of the exponential phase (OD_600_ ≈ 1.5 for control group and 2.0 for stressed group), cells were harvested in order to isolate RNA.

### RNA extraction and cDNA synthesis

Total RNA was extracted by following protocol of Rivas and Vizcaino [63]. Three independent biological repeats were conducted for each treatment and the extracted RNA samples were mixed together for each treatment. Total RNA was treated with RNase-free DNaseI according to the manufacturer’s recommendations (Ambion, USA) and incubated in 37°C for 1 hour. The purified RNA was checked by amplifying 16 s rDNA using polymerase chain reaction (PCR) (35 cycles). Then, rRNA was removed using Ribo-ZeroTM rRNA Removal Kit according to the manufacturer’s recommendations (Epicentre, USA). The quantity and purity of the purified RNA was assessed using NanoDrop ND-1000 and Agilent 2100 Bioanalyzer. The A260/A280 ratio of the two samples was ≥2.0.

To generate a cDNA library, the total RNA removed 16S and 23S rRNA was fragmented into small pieces at a specific temperature and then the random premier with biotin and illumina primer (Oligonucleotide sequences © 2006–2010 Illumina, Inc) was used to anneal with the fragmented mRNA. The first strand of cDNA was then synthesized using reverse transcriptase. Finally, the double strand cDNA was synthesized by PCR with illumina primer.

### Illumina sequencing and data analysis

The cDNA between 300 and 500 bp was obtained by gel extraction. The extracted cDNA was amplified using TruSeq PE Cluster Kit (Illumina,USA) and then sequenced on Illumina Hiseq2000 to generate 100-bp single-end reads.

The clean reads were got by removing the low quality sequences and then mapped to the reference genome using short oligonucleotide alignment program (SOAP) [64]. RPKM (number of reads per kilobase of exon region per million mapped reads) was used to normalize the expression level of genes [65]. The difference of gene expression between two samples were counted using MA-plot-based method with Random sampling model (MARS) in DEGseq software package [66]. The differentially expressed genes were considered to be induced or repressed if the normalized fold change (log2(fold change)) >1 and the false discovery rate (FDR) <0.001[67].

Reverse Position Specific BLAST (RPS-BLAST) program was used to obtain the COG items (clusters of orthologous groups) of the genes [19]. The KO (KEGG Orthology) codes of genes were obtained using bidirectional best hit method (BBH) aligned with Kyoto Encyclopedia of Genes and Genomes database (KEGG) [21]. The pathway enrichment analysis was conducted using hypergeometric distribution method. The results of the RNA sequencing experiments have been submitted to GEO database with an accession number of GSE57306.

### Validation of RNA-Seq data by RT-qPCR

The expression levels of 14 representative genes were examined by RT-qPCR to validate the RNA-Seq data (Table 4). The primers for RT-qPCR were designed using Primer 3 (Additional file 7) [68]. These primers were pruned against the genome of *M. alhagi* to ensure their specificity. The sizes of PCR products were confirmed by electrophoresis in 2% agarose gel. The RNA extraction was conducted the same as in RNA extraction and cDNA synthesis. Moreover, the cDNA synthesis was conducted using PrimeScriptTM RT reagent Kit with gDNA Eraser (Takara Japan). Quantitative real-time PCR was conducted on BioRad CFX96 Real-Time System using SYBR® Premix Ex Taq (Takara Japan). For each gene/sample combination, three replicate reactions were carried out. In addition, the 16 s rDNA gene was chosen as a reference gene.

**Table 4:** **Genes used to validate the RNA-Seq data**

**GI number**	**Expression state**	**Gene annotation**
359249574	DOWN	ClpA/B-type protease
359249577	DOWN	type VI secretion protein, evpb/vc_a0108 family
359250755	UP	hypothetical protein MAXJ12_26333
359250756	UP	YadA domain-containing protein
359251989	UP	Na+/solute symporter (Ssf family) protein
359252234	DOWN	chaperonin GroEL
359252475	DOWN	lytic transglycosylase
359252632	DOWN	PrkA family serine protein kinase
359252964	UP	AraC type helix-turn-helix- domain containing protein
359254159	UP	glycine betaine transporter periplasmic subunit
359255162	DOWN	hypothetical protein MAXJ12_06665
359255790	UP	NodF
359256067	UP	S-adenosylmethionine synthetase
359256447	UP	Na+/H+ antiporter NhaA

### Gene knockout mutant construction and complementation tests

Bacterial strains and plasmids used in gene knockout mutant construction and complementation tests are listed in (Additional file 8). Primers used in this part are listed in (Additional file 9). For gene knockout construction, we firstly designed four primers to amplify the upward and downward fragments of each target gene. Secondly, we digested the fragments and pk18*mobsacB* plasmid with proper restriction enzymes using standard protocols. Thirdly, we conducted that the prepared plasmids and fragments ligation reaction using T4 ligase and transformed the ligation products into *Escherichia coli* DH5α component cells. Finally, triparental mating procedure was conducted to transforme the plasmids from *E. coli* to *M. alhagi* as described previously [69]. Briefly, the DH5α strains containing the plasmids constructed, the MM249 strains containing helper plasmid pRK2013 and wild type *M. alhagi* mixed together and cultured on TY plate for three days. Then, SM plates containing kanamycin (50 ug/ml) were used to isolate the single exchange of *M. alhagi* mutants. The TY plates containing sucrose (5 g/100 ml) were used to isolate the double exchange mutants. The obtained geneknock mutants were verified by PCR. For complementation mutant strain construction, we designed primers to amplify the entire open reading frame of each gene. The folloing producers were similar to the gene knockout mutant construction. The difference of the complementation mutant construction was that the plasmid used was pBBR1MCS-5 and the antibiotic uesd was gentamicin. The strains containing the complementation plasmids were selected on SM plates containing gentamicin (50 ug/ml) and validated by colony PCR.

### Growth analysis under salt stress

Wild type of *M. alhagi* and the gene knockout mutants were cultured to OD ≈ 0.5 in TY broth medium. 5 ul suspensions of the fourstrains was inoculated into 24 well culture plate (Cyagen Biosciences Inc. USA) containing 1 ml TY broth medium with and without 0.4 M NaCl. For the complementation tests, the TY borth medium with and without 0.4 M NaCl containing gentamicin (50 ug/ml) was used to obtain the growth curve. The OD_600_ of the plates were detected every 12 hours using Epoch (Bioteck, USA). For measuring the growth curves of *M. alhagi* under different salt concentrations, the inoculum was cultured to OD ≈ 0.1 and then 1 ml of the inoculum was inoculated into 100 ml TY broth medium with different salt concentrations (0, 0.1, 0.2, 0.3, 0.4 and 0.5 M NaCl). The flasks were incubated in a shaker at 28°C and agitated at 120 rpm. The OD_600_ of the cultures were detected every 12 hours using Lambda 35 UV/VIS Spectrometer (PerkinElmer, USA).

## Additional file

Additional file 1
**Growth curve of XJ12-2 in TY medium under different salt stress.** Optical density at 600 nm (OD_600_) was used to monitor the bacteria growth. The mean values of three independent experiments are shown and the error bars represent the standard deviation. The arrows point out the sample collection time. Additional file 2
**The number of genes in each COG categories.**
Additional file 3
**Overrepresented functional KO terms of down-regulated genes.**
Additional file 4
**Overrepresented functional KO terms of up-regulated genes.**
Additional file 5
**Representative DEGs involved in cell growth, protein synthesis and energy production.**
Additional file 6
**DEGs involved in protein secretion systems and lytic transglycosylases.**
Additional file 7
**Primers used in RT-qPCR.**
Additional file 8
**Bacteria strains and plasmids used in this study.**
Additional file 9
**Primers used in mutant construction.**
