# Supplementary figures and images for: Global transcriptome analysis of Mesorhizobium alhagi CCNWXJ12-2 under salt stress
Source: BMC Microbiol. 2014 Dec 24;14:319. doi: 10.1186/s12866-014-0319-y (PMC4302635; doi:10.1186/s12866-014-0319-y)

## Slide 1
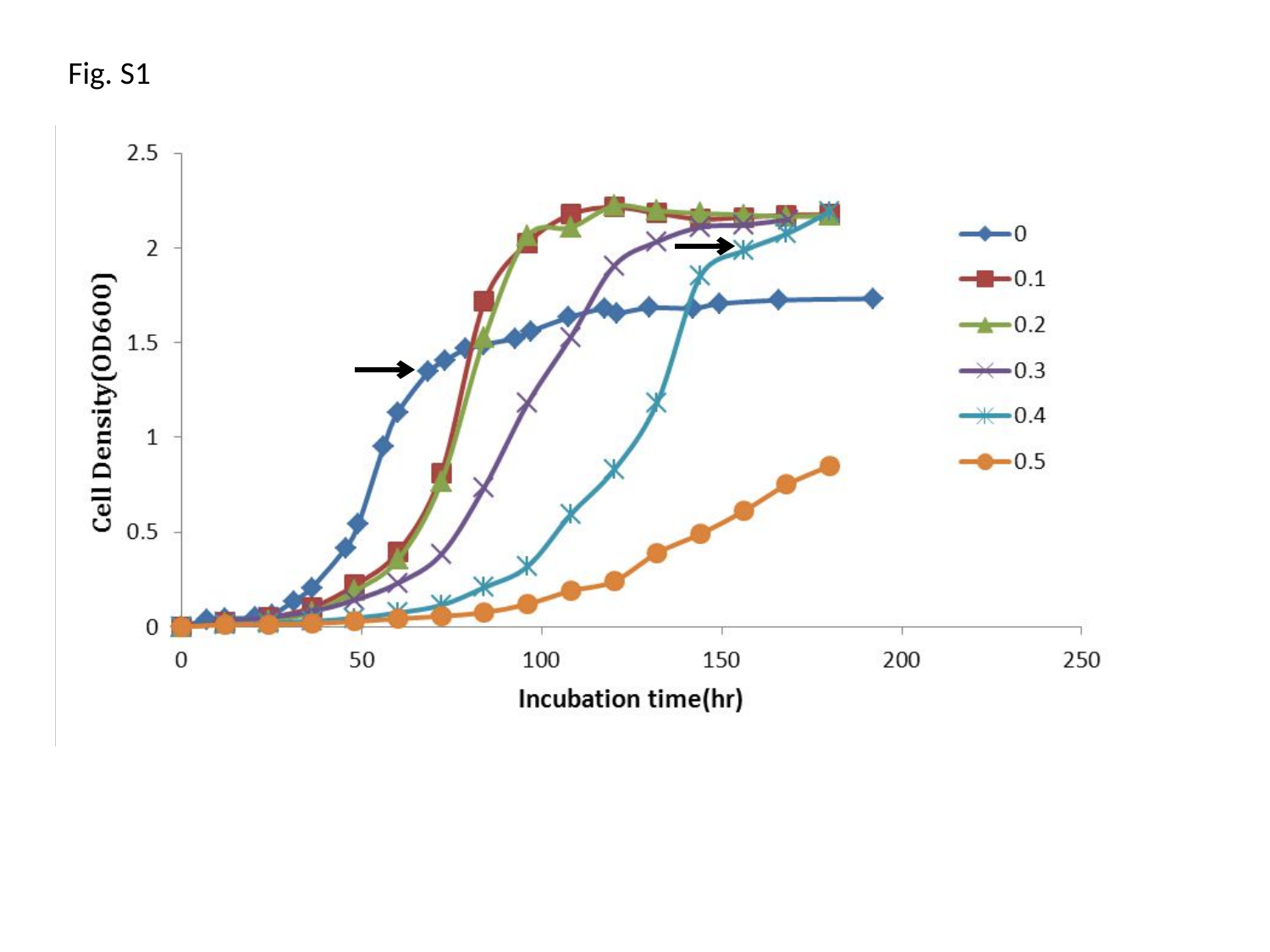

Fig. S1

Supplement: Additional file 1 — Growth curve of XJ12-2 in TY medium under different salt stress. Optical density at 600 nm (OD600) was used to monitor the bacteria growth. The mean values of three independent experiments are shown and the error bars represent the standard deviation. The arrows point out the sample collection time. [file 12866_2014_319_MOESM1_ESM.pptx]
